# Soluble CD40 ligand-activated human peripheral B cells as surrogated antigen presenting cells: A preliminary approach for anti-HBV immunotherapy

**DOI:** 10.1186/1743-422X-7-370

**Published:** 2010-12-23

**Authors:** Chao Wu, Yong Liu, Qi Zhao, Guangmei Chen, Junhao Chen, Xiaomin Yan, Yi-Hua Zhou, Zuhu Huang

**Affiliations:** 1 Department of Infectious Diseases, Nanjing Drum Tower Hospital, Nanjing University Medical School, 321 Zhongshan Road, Nanjing, Jiangsu, PR China; 2 Department of Laboratory Medicine, Nanjing Drum Tower Hospital, Nanjing University Medical School, 321 Zhongshan Road, Nanjing, Jiangsu, PR China; 3 Department of Infectious Diseases, First Affiliated Hospital, Nanjing Medical University, Nanjing, PR China

## Abstract

**Background:**

We aimed to clarify whether soluble CD40 ligand (sCD40L) activated B cells may be loaded with HBcAg18-27 peptide and served as antigen-producing cells (APCs) to induce HBV-specific cytolytic T lymphocytes (CTLs).

**Results:**

Human B cells could be cultured in the presence of sCD40L up to 54 days, and the proportion of B cells in the S phase increased from 0% to 8.34% in the culture. The expression of CD80, CD86, major histocompatibility complex (MHC) classes I and II molecules on the sCD40L-activated B cell was significantly increased after long-time culture. Cytometry and fluorescence microscopy showed that more than 98% sCD40L-activated B cells were loaded by the HBcAg peptide. Furthermore, the peptide-pulsed activated B cells could induce HBcAg18-27 specific CTLs.

**Conclusions:**

Our results demonstrate that sCD40L-activated B cells may function as APCs and induce HBV-specific CTLs.

## Background

Efficient antigen presentation by antigen presenting cells (APCs) is critical for inducing T-cell mediated immunity *in vivo *[1,2]. Dendritic cells (DCs), activated macrophages, and activated B cells are all capable of presenting antigen peptides. DCs are considered to be highly efficient at antigen capture, processing, and migration [3]. Therefore, DCs have been used to generate antigen-specific T cells for immunotherapy [4-6].

Recently, it has been demonstrated that B cells may function as APCs [1,7] in addition to the essential role in the humoral immune response. Banchereau *et al *first reported the "CD40 system" [8], and suggested to use CD40 ligand (CD40L) stimulated B cells as an alternative or complementary APC. The CD40L-activated B cells may be continually expanded and the B cells significantly up-regulate the expression of major histocompatibility complex (MHC) class I and class II and induce the expression of CD80 and CD86. Antigen-specific CD40L-activated B cells may efficiently endocytose and present antigens, such as protein, RNA, and cDNA, to prime primary T cells and boost robust memory T-cell responses [9]. More importantly, activated B cells may also prime naive T-cell responses against neoantigens *ex vivo *as DCs do [9]. Thus, the activated B cells may serve as cellular adjuvants to present antigens *in vivo *[10].

The mechanism of chronic hepatitis B virus (HBV) infection remains unclear. Previous studies have suggested that functional impairment of DCs may mediate suppression of viral-specific T-cell immune response, resulting in viral persistence in the chronic HBV infection [11-13]. As another type of important APCs, B cells may also function as primary APC in CHB infection [14]. However, little is known whether CD40L-activated B cells may present HBV antigen to T cells.

In this study, we set up an effective culture method for long-term maintenance of B cells *in vitro*, in which the B cells are activated by human soluble CD40L (sCD40L). Furthermore, we provide evidence that the activated B cells may serve as APCs to present core peptide of HBV to cytolytic T lymphocytes (CTLs).

## Results

### Proliferation of B cells activated by sCD40L

**Figure 1:**
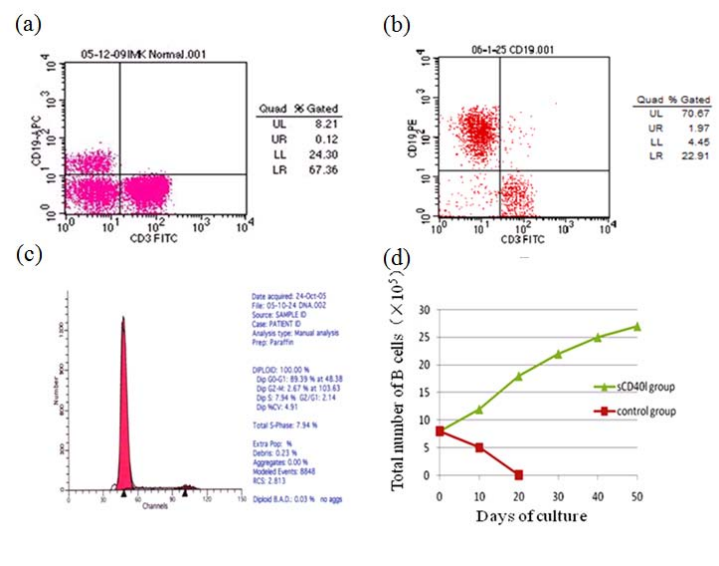
**Proliferation profile of sCD40L-activated B cell**. (a) The percentage of B cells in the PMBCs. It was about 8.21% of total cell population at the initiative culture. (b) The percentage of B cells increased up to 70.67% of the total cells as cultured for 48 days. (c) Cells were stained for DNA content with PI-pretreatment and analyzed of the cell cycle by flow cytometry. The y-axis shows relative cell number and the x-axis shows DNA content. sCD40L-stimulated B cells accumulated in the phase S. (d) B cell counts in the presence or absence of sCD40L. The x-axis shows days of cell culture and the y-axis shows the number of the B cells. sCD40L-stimulated cells increased in number but the non-stimulated cells decreased.

As a terminal cell type, B cells in peripheral blood mononuclear cells (PBMCs) can usually be cultured for 2-3 weeks only, which limits the application of B cells as APCs. To prolong the culture period, we added sCD40L into the culture of PBMCs, which resulted in the prolonged culture period as long as 54 days in the presence of sCD40L. FACS analyses showed that the percentage of B cells in the culture increased significantly over the time, and B cells accounted for about 80% of the total PBMCs when the cells were cultured for 54 days (Figure 1a and 1b). In contrast, the PBMCs cultured in the absence of sCD40L contained no B cells analyzed by cytometry 20 days after culture (Figure 1d).

Additionally, cell cycle profiles analyzed by cell cycle distribution indicated that the G1 phase decreased from 99.87% on day 3 to 88.92% on day 45, concomitant with an increase in cells in the S phase from 0% to 8.34% and the G2/M phase from 0.13% to 2.74% (Figure 1c). However, no decrease in the sub-G1 cells was detected in the culture without sCD40L. The results demonstrated that the B cells were able to re-enter the S phase and proliferate in the presence of sCD40L compared with the cells cultured in the absence of sCD40L. Total number of B cells in the presence of sCD40L increased from 8.84 × 10^5 ^to 8.61 × 10^6^, while the number of B cells in the absence of sCD40L was decreased (Figure 1d). Taken together, in accordance with the previous reports [15], our data demonstrated that B cells may proliferate for significantly long period of time in the presence of sCD40L. After completion of the experiments on the above donor, we repeated all the culture process from another donor's sample; the results were comparable or almost same.

### Increased expression of CD80, CD86, MHC classes I and II on cell surface of sCD40L-activated B cells

**Figure 2:**
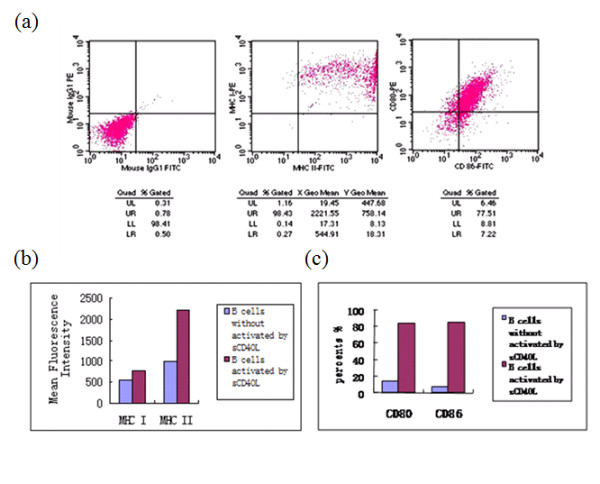
**Detection of the expression of CD80, CD86, and MHC I and II on sCD40L-activated human B cells as surrogates of APC**. (a) The expression of surface molecules MHC-I, MHC-II, CD80, and CD86 on B cells were examined by gating CD19 positive cells by FACS analysis. (b) The expression of MHC-I and MHC-II on B cells was expressed as the mean fluorescence intensity. (c) The expression of CD80 and CD86 on B cells was expressed as the percents in all B cells.

Previous studies demonstrate that human B cells isolated from peripheral blood may be activated and the expression levels of CD80, CD86, MHC classes I and II molecules on the cell surface is efficiently up-regulated by infection with Epstein-Barr virus or co-culture with mitogen-induced cells transfected with the human CD40L [16,17], and the B cells may serve as APCs and induce specific CTLs. However, the previous culture systems introduced the extraneous source germ cells or virus and limited the further clinical application researches. In our experiment, we cultured B cells with recombinant human (rh) sCD40L and the cells could be continuously expanded in long term culture. To investigate whether the activated B cells may serve as APC, we detected the expression of costimulatory molecules, including CD80, CD86, MHC classes I and II on the cell surface by flow cytometry. Figure 2 presents that the levels of these molecules on the sCD40L-activated B cells were significantly increased. In contrast, the levels of all above cell surface molecules were very low on the B cells before activation. Thus, the results suggest that the B cells activated by sCD40L may have the function of APCs.

### Visualization of antigen delivery to sCD40L-activated B cells

**Figure 3:**
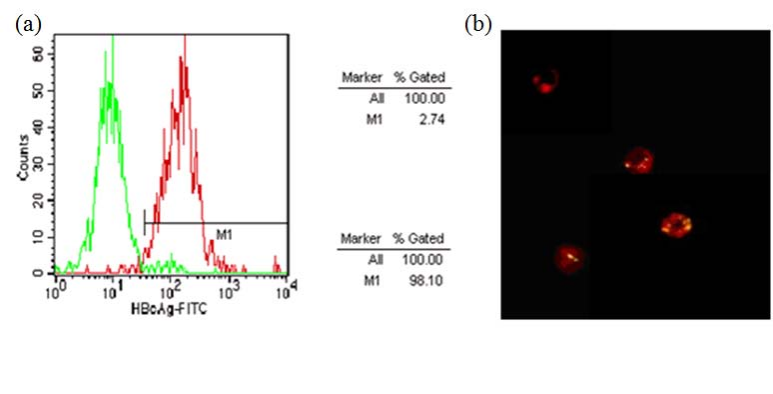
**HBV core peptide was loaded on sCD40L-activated B cells**. As negative control, auto fluorescence of CD40-B cells and isotype control are shown. (a) To gate CD19 positive cells by FACS analysis, the green curve was isotype control and the red curve was B cells specific binding of HBV core peptide. The y-axis shows relative cell number and the x-axis shows the fluorescence intensity of the cells. (b) To observe the B cells specific binding of HBV core peptide by fluorescence microscope, the red fluorescence located at B cell surface was CD19-PE and the yellow fluorescence (superimposition of the green FITC fluorescence and the red CD19-PE becomes yellow) located in cytoplasm was HBV core protein (FITC-FLPSDFFPSV).

Since the expression levels of CD80, CD86, MHC classes I and II molecules on the B cell surface were significantly increased after long-time culture with sCD40L, sCD40L-activated B cells may have the function of antigen presentation. To clarify whether this is true or not, we cultured the cells in the presence of a fluorochrome-labeled peptide, which was derived from the core protein of HBV. Cytometry analysis showed that more than 98% of sCD40L-activated B cells had the green fluorescence (Figure 3a), indicating that there was HBV core peptide in the cells or on the surface of the cells and the B cells might be loaded by the HBV core peptide. We further observed the cells under fluorescence microscope and found that the red fluorescence located at B cell surface was CD19-PE and the green fluorescence (superimposition of the green FITC fluorescence and the red CD19-PE becomes yellow) located in cytoplasm was HBV core peptide. The activated B cells showed strong fluorescence after peptide pulsing at concentrations even lower than 25 μg/mL (Figure 3b). All of the above results indicate that the sCD40L-activated B cells may be loaded with the HBV core peptide.

### The result of the HBcAg18-27 specific CTLs

**Figure 4:**
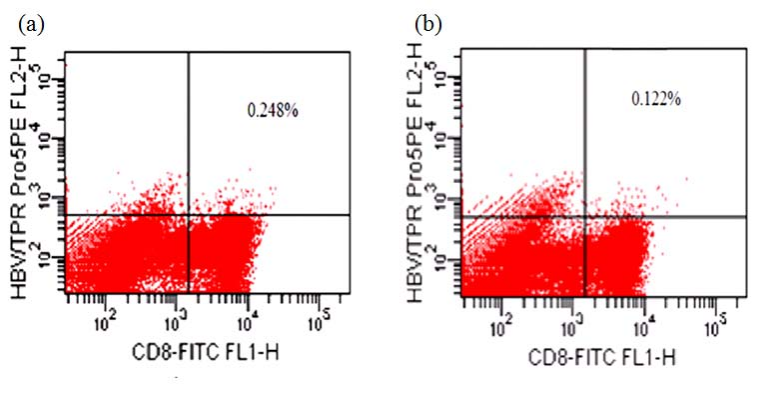
**Pentamer analysis of induction of CTL responses against peptides of HBcAg 18-27 by CD40-B cells**. (a) 0.248% of the T cells were induced to be HBcAg18-27 specific CTLs. (b) While such CTLs were only 0.122% in the absence of activated B cells.

To investigate whether the sCD40L-activated B cells may present the HBV core peptide to T cells and induce specific cytotoxic T cell responses, we co-cultured the autologous T cells and sCD40L-activated B cells loaded by HBV core peptide, and then detected the CTL responses against peptides of HBcAg 18-27 by pentamer analysis. FCM analyses showed that 0.248% of the T cells were induced to be HBcAg18-27 specific CTLs, while such CTLs were only 0.122% in the absence of activated B cells (Figure 4). Hence, the sCD40L-activated B cells may function as APCs and induce HBV-specific CTL.

## Discussion

Recently, DC-based immunotherapy has gained a lot of interest in clinical immunology [18]. The high efficiency of DC vaccines has been proved in anti-tumor and anti-virus immunological treatment. However, the DCs constitute only 0.1-0.5% of human PBMC. Technical difficulty and high cost to obtain sufficient number of highly enriched mature DCs have limited the clinical applications of dendritic cell vaccine.

In this study, we used recombinant human GMP-quality trimeric soluble CD40L to activate PBMC-derived B cells. We established and optimized the culture system for CD40L-B cells, which allows B cell activation and proliferation without the contamination from extraneous source germ cells and genes. It was noted that rh sCD40L-activated B cells could be cultured *in vitro *for up to 54 days under the culture condition. At the same time, B cells significantly up-regulated MHC class I and class II expression and induced expression of CD80 and CD86 after activated by rh sCD40L. Unlike some recent studies, in which B cells were co-cultured with NIH3T3 cells or other tumor cells which steadily express CD40L [8,19], our results demonstrated that B cells could be activated and expanded for prolonged period of time. While previous culture system has introduced the extraneous source germ cells and has limited the further clinical application researches, the accomplishment of present work may be taken as an alternative way to activate primary human B cells *in vitro*.

Because sufficient expression of MHC and costimulatory molecules is closely associated with APC function, phenotypic analysis of the cell surface molecules is applied as a reliable surrogate readout for APC function of B cells [20]. Moreover, costimulatory molecules CD80 and CD86 expressed on APCs are required for the development of T cell responses, which play important roles in the differentiation of Th1- or Th2-phenotypes [21]. CD80 and CD86 expressed on the surface of antigen-presenting cells interact with CD28 and cytotoxic T lymphocyte antigen-4 expressed on activated T cells. Interaction between CD80/86 and CD28 mediates critical T cell stimulatory signals, which may cause T cells to stably secrete IL-2 and other cytokines, and maintain T cell survival [22]. Our experiments demonstrate that the rh sCD40L may activate the B cells from PBMCs, and induce a strong up-regulation of those surface molecules associated with antigen processing on human B lymphocytes. Functionally, our study has moved a step forward to demonstrate that 98% of sCD40L-activated B cells may be loaded with HBcAg18-27 peptide. Furthermore, after co-cultured with the peptide-pulsed CD40L-B cells, more HBcAg18-27 specific CTLs were detected in autologous PBMCs. All of these results indicate that the CD40L-B cells have the characteristics of APCs.

The weakness of this study is that all data were just derived from 2 healthy donor samples. However, we performed the experiments separately, not at the same time, i.e., we cultured the B cells from one donor and did the relevant experiments, and then we repeated all the experiments using another donor's sample, the results were comparable or almost same. Thus, we consider that the long-term culture system for B cells developed in this work is reproducible and the data in the present work are sufficient to support our conclusion.

## Conclusions

In summary, the present study has established an approach for a long term culture of human B cells from PBMCs under the stimulation of sCD40L and the sCD40L activated B cells may serve as APCs. Furthermore, antigen presenting activity of sCD40L-acitvated B cells was evidenced by antigen-loading and the induction of HBcAg18-27 specific CTLs in autologous PBMCs. Thus, CD40L-activated B cells may be used as a potential source of APCs for adoptive immunotherapy for chronic HBV infection.

## Methods

### Blood samples

Peripheral blood (30 ml) was obtained by venipuncture from two healthy donors with HLA-A2+. The donors gave the consent and all the experiments were approved by the Ethics Committee of Nanjing Drum Tower Hospital, Nanjing University Medical School, in accordance with guidelines of the Nation Health and Medical Research Council of China. PBMCs were isolated by Ficoll-density centrifugation, rinsed twice with iscoves modified Dulbecco medium (IMDM) (Gibco BRL, Carlsbad, USA). Approximately 3 × 10^7 ^PBMCs were acquired; 1.6 × 10^7 ^PBMCs were further cultured for preparing activated B cells by sCD40L, and the surplus cells were kept in cryopreservation fluid and frozen in liquid nitrogen for pentamer analysis.

### Activation of B cells by sCD40L

PBMCs were plated in wells of 6-well plates (8 × 10^6 ^cells in 4 ml per well) in IMDM supplemented with 10% human serum with blood type AB, rh IL-4 (2 ng/ml) (R&D Systems, Minneapolis, USA), insulin (5 μg/ml) (Roche, Mannheim, Germany), cyclosporin A (CsA) (5.5 × 10^-7 ^M) (Novartis, Basel, Switzerland), transferrin (50 μg/ml) (Eappel, USA), and gentamicin (15 μg/ml) (Lukang, Shandong, China). Good medical practice (GMP)-quality trimeric rh soluble CD40L (rh sCD40L) (R&D Systems, Minneapolis, USA) was added to a final concentration of 2 μg/ml. The culture was maintained by replacing half medium with the same medium, in which CsA and sCD40L were freshly added. B cells that were cultured in the same medium except that sCD40L were omitted served as controls.

### Cell proliferation assay

Cell proliferation was determined by analysis of cell cycle distribution with flow cytometry using Cycle Test Plus DNA Reagent kit (Becton Dickinson, San Jose, USA) as previously described [23]. In brief, the cultured cells (1 × 10^5^) were collected and digested by trypsin, followed by adding trypsin inhibitor and RNAase, then mixed with propidium iodide in the dark condition at 4°C. The cells were then subjected to run on the BD FACSCanto flow cytometer (BD Biosciences, CA, USA). Based on the intensity of the fluorescent light signal emitted by the DNA-binding dye, the cell populations were located in four distinct phases, which may be recognized in static phase (G0/G1), DNA synthesis phase (S), and DNA mitosis phase (M), respectively [23].

### Assay for cell surface molecules

The cell surface molecules, including CD86, CD80, MHC classes I and II, on sCD40L-activated B cells were analyzed by flow cytometry. In brief, 1 × 10^6 ^PBMCs in 100 μl PBS were rinsed twice with PBS containing 2% FBS and divided into 3 tubes: anti-CD19-APC, anti-CD86-FITC, and anti-CD80-PE were added into the first tube, anti-CD19-APC, anti-MHC-II-FITC and anti-MHC-I-PE were added into the second tube, and in the third tube, negative isotype control staining reactions were in parallel performed with a saturating concentration of irrelevant mouse IgG1-FITC and IgG1-PE. All above seven antibodies were purchased from BD Biosciences (BD PharMingen, San Diego, CA). After incubated for 20 min and rinsed with PBS, the cells were fixed with 1% paraformaldehyde in PBS. FACS analysis was performed on the BD FACSCanto flow cytometer. B cells were gated on CD19-positive cells for analyzing the cell surface molecules.

### Analysis of peptide pulsing

HBV derived peptides were used for peptide pulsing study as previously described [24,25] with modifications. The HLA-A*0201-binding peptide of HBV core 18-27 (HBcAg 18-27, Phe-Leu-Pro-Ser-Asp-Phe-Phe-Pro-Ser-Val, FLPSDFFPSV), a confirmed HLA-A 2.1-restricted CTL epitope and derived from the HBV core protein [26], was obtained from Sangon (Shanghai, China). For the MHC class I experiments, the peptide was conjugated by FITC at the first residue of the N-terminus. CD40L-activated B cells were harvested from culture, washed 3 times, and resuspended in serum-free IMDM at 2 × 10^5 ^cells/ml and seeded into 24-well plates (1 ml/well). After incubation with the FITC-conjugated peptide for 12-18 hours, the cells were harvested, washed, and resuspended in PBS. Fluorescence analysis was performed immediately on the BD FACSCanto flow cytometer for detecting the ratio of B cells specific binding of HBV core peptide. The residual cells were observed by fluorescence microscope (Zeiss, Goettingen, Germany) for determining the location of HBV core peptide in B cells. B cells were also incubated with no FITC-conjugated peptide under the same condition for pentamer analysis.

### Pentamer analysis

On day 45 of B cells culture from one donor, the CD40L-activated B cells were used as the source of APCs for stimulation of autologous T cells. In brief, the frozen PBMCs were rapidly thawed in a 37°C water bath and then cultured at a concentration of 2 × 10^6 ^cells/ml in RPMI 1640 supplemented with 10% fetal bovine serum and rhIL-2 (10 ng/ml) (R&D Systems). On day 4, the cultures were replaced with the same medium, followed by co-culture with the peptide-pulsed CD40L-activated B cells. After 4 days, the cells were harvested, washed, and mixed with both anti-CD8-FITC and PE-labeled HLA-A2 pentamer complexes against the HBV core 18-27 peptide (ProImmune, Oxford, UK). The cells were resuspended in 500 μl of PBS and two-colour analysis was performed by BD FACSCanto flow cytometer. For each analysis, 100 000 events were acquired. PBMCs that were cultured in the same medium omitted peptide-pulsed CD40-B served as controls.

## Competing interests

The authors declare that they have no competing interests.

## Authors' contributions

CW performed the experiments, analyzed the data, and drafted the manuscript. YL, QZ and GC performed the experiments and analyzed the data. XY and JC collected the samples and assisted in the performance of the experiments. YHZ interpreted the data and revised the manuscript. ZH designed the study, interpreted the data and critically revised the manuscript. All authors read and approved the final manuscript.
